# Regioselective Nucleophilic Aromatic Substitution: Theoretical and Experimental Insights into 4-Aminoquinazoline Synthesis as a Privileged Structure in Medicinal Chemistry

**DOI:** 10.3390/molecules29246021

**Published:** 2024-12-20

**Authors:** Maria Letícia de Castro Barbosa, Pedro de Sena Murteira Pinheiro, Raissa Alves da Conceição, José Ricardo Pires, Lucas Silva Franco, Carlos Mauricio R. Sant’Anna, Eliezer J. Barreiro, Lídia Moreira Lima

**Affiliations:** 1 Faculty of Pharmacy, Department of Pharmaceutical Sciences, Federal University of Juiz de Fora, Juiz de Fora 36036-900, MG, Brazil; 2 Laboratory of Evaluation and Synthesis of Bioactive Substances (LASSBio), Institute of Biomedical Sciences, Federal University of Rio de Janeiro, Rio de Janeiro 21941-902, RJ, Brazil; pedro.pinheiro@icb.ufrj.br (P.d.S.M.P.); silvafrancolucas@gmail.com (L.S.F.); santanna@ufrrj.br (C.M.R.S.); ejbarreiro@ccsdecania.ufrj.br (E.J.B.); 3 National Institute of Science and Technology in Drugs and Medicines (INCT-INOFAR), Rio de Janeiro 21941-902, RJ, Brazil; 4 Faculty of Pharmacy, Federal University of Rio de Janeiro, Rio de Janeiro 21941-902, RJ, Brazil; raissa.conceicao9@gmail.com; 5 Institute of Medical Biochemistry Leopoldo de Meis (IBqM), Federal University of Rio de Janeiro, Rio de Janeiro 21941-902, RJ, Brazil; murari@bioqmed.ufrj.br; 6 Institute of Chemistry, Federal Rural University of Rio de Janeiro, Seropédica 23970-000, RJ, Brazil

**Keywords:** aminoquinazoline, density functional calculations, NMR spectroscopy, nucleophilic substitution, regioselectivity, medicinal chemistry

## Abstract

The 4-aminoquinazoline scaffold is a privileged structure in medicinal chemistry. Regioselective nucleophilic aromatic substitution (S_N_Ar) for replacing the chlorine atom at the 4-position of 2,4-dichloroquinazoline precursors is well documented in the scientific literature and has proven useful in synthesizing 2-chloro-4-aminoquinazolines and/or 2,4-diaminoquinazolines for various therapeutic applications. While numerous reports describe reaction conditions involving different nucleophiles, solvents, temperatures, and reaction times, discussions on the regioselectivity of the S_N_Ar step remain scarce. In this study, we combined DFT calculations with 2D-NMR analysis to characterize the structure and understand the electronic factors underlying the regioselective S_N_Ar of 2,4-dichloroquinazolines for the synthesis of bioactive 4-aminoquinazolines. DFT calculations revealed that the carbon atom at the 4-position of 2,4-dichloroquinazoline has a higher LUMO coefficient, making it more susceptible to nucleophilic attack. This observation aligns with the calculated lower activation energy for nucleophilic attack at this position, supporting the regioselectivity of the reaction. To provide guidance for the structural confirmation of 4-amino-substituted product formation when multiple regioisomers are possible, we employed 2D-NMR methods to verify the 4-position substitution pattern in synthesized bioactive 2-chloro-4-aminoquinazolines. These findings are valuable for future research, as many synthetic reports assume regioselective outcomes without sufficient experimental verification.

## 1. Introduction

**Scheme 1:**
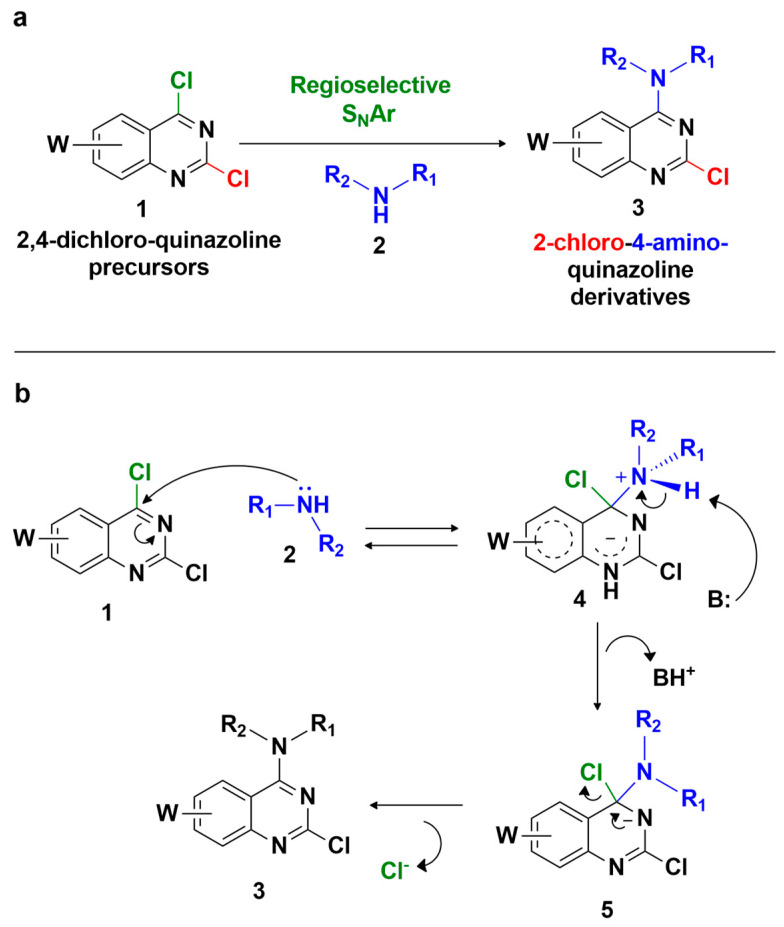
(**a**) General scheme for regioselective nucleophilic aromatic substitution (S_N_Ar) reaction from 2,4-dichloro-quinazoline precursors and primary or secondary amine nucleophiles; (**b**) intermediates involved in the regioselective S_N_Ar reaction from 2,4-dichloro-quinazolines (**1**) and primary or secondary amine nucleophiles (**2**), providing the corresponding 2-chloro-4-aminoquinazoline derivatives (**3**).

Regioselective nucleophilic aromatic substitution (S_N_Ar) at the 4-position chlorine of 2,4-dichloroquinazoline precursors by amine nucleophiles is well documented and extensively studied in the scientific literature (Scheme 1) [1,2,3,4,5,6,7,8,9,10,11].

**Figure 1:**
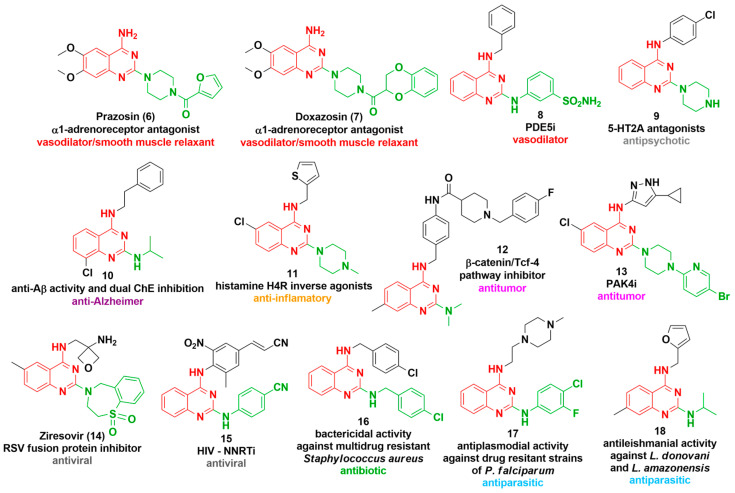
Bioactive 4-aminoquinazolines (**6**–**18**) described as drugs or as drug candidates presenting vasodilator, smooth muscle relaxant, antipsychotic, anti-Alzheimer, anti-inflammatory, antitumor, antiviral, antibiotic, and antiparasitic pharmacological properties. The 4-aminoquinazoline scaffold is highlighted in red, and the second amino substituent of the 2,4-diaminoquinazolines is indicated in green. Aβ = amyloid beta peptide; ChE = cholinesterase; H4R = histamine H4 receptor; 5-HT2A = serotonin 5-HT2A subtype receptor; PAK4i = p21 activated kinase 4 inhibitor; PDE5i = phosphodiesterase 5 inhibitor; Tcf-4 = transcription factor 4; HIV = human immunodeficiency virus; NNRTi = non-nucleoside reverse transcriptase inhibitor; RSV = respiratory syncytial virus.

This chemical transformation is widely used in the synthesis of novel bioactive compounds, specifically 2-chloro-4-aminoquinazolines [2,3,5] and/or 2,4-diaminoquinazolines [1,6,7,8,10,11,12,13,14,15,16,17,18], for various therapeutic applications. These include vasodilation (**6**–**8**) [12,18,19], antipsychotic (**9**) [15], anti-Alzheimer’s (**10**) [8,16], anti-inflammatory (**11**) [13], antitumor (**12**,**13**) [2,3,4,5,14,17,20,21], antiviral (**14**,**15**) [9,22], antibiotic (**16**) [6,23,24], and antiparasitic (**17**,**18**) [7,10,11,25] treatments, as demonstrated by several pertinent examples presented in Figure 1.

A privileged structure can be defined as a molecular framework that offers effective ligands for a range of biological targets [26]. Through careful and rational structural modifications, the plethora of different pharmacological activities exhibited by the 4-aminoquinazoline derivatives [11,27] allows this structural moiety to be characterized as a privileged structure in medicinal chemistry. This makes it a highly valuable scaffold for the design and development of novel drug candidates (Figure 1).

As shown in Table 1, numerous studies in the scientific literature report S_N_Ar reactions starting from 2,4-dichloroquinazoline precursors (**1a**–**k**) with a variety of nucleophiles, including anilines [2,3,5,10], benzylamines [6,7,14,20,23], and primary or secondary aliphatic amines [1,2,4,6,7,8,9,10,11]. These reactions, using different methodologies and diverse reaction conditions, consistently demonstrate regioselectivity for substitution at the 4-position of the quinazoline ring, resulting in the formation of 2-chloro-4-aminoquinazoline derivatives (see Scheme 1).

The regioselective S_N_Ar examples summarized in Table 1 show that reaction time can range from minutes [4] to several hours [2,5,17], depending on the reaction conditions, the reactivity of the quinazoline, and the nucleophilicity of the amine. Typically, aromatic, benzylic, and aliphatic primary or secondary amines are employed as nucleophiles. Additionally, the substitution pattern and electronic properties of the electrophilic quinazoline nucleus can influence S_N_Ar regioselectivity, as suggested by the literature on related heterocycles, such as 2,4-dichloropyrimidines [28]. Table 1 also highlights both non-functionalized (**1a**) and variously functionalized (**1b**–**k**) 2,4-dichloroquinazoline precursors, with electron-donating and electron-withdrawing groups, that still maintain 4-position regioselectivity in S_N_Ar. Key examples include commonly used precursors such as 2,4-dichloroquinazoline (**1a**) [1,3,10,16], 6,7-dimethoxy-2,4-dichloroquinazoline (**1b**) [2,5,7,11], and 2,4,6-trichloroquinazoline (**1f**) [8,13,21], all of which demonstrate consistent regioselectivity for substitution at the 4-position.

Regarding the polarity of the reaction solvent, polar solvents are often favored, since this chemical transformation involves the generation of charged intermediates (Scheme 1b), which can be effectively stabilized by electrostatic interactions within the reaction medium. Previously described examples included the use of polar protic solvents such as methanol [9], ethanol [2,5,8,13,16,20], and 2-propanol [2,5], as well as polar aprotic solvents like tetrahydrofuran (THF) [2,4,5,15,17,18,23], acetonitrile [7,11], and dimethylformamide (DMF) [21]. Notably, THF and ethanol emerge as the most frequently utilized aprotic and protic polar solvents, respectively. Additionally, a mixture of polar protic (water) and aprotic (THF) solvents represents an interesting alternative option [3,6,10,24,25].

Typically, the reaction is conducted in the presence of an inorganic base, commonly sodium acetate (NaOAc) [3,6,10,24,25], or an organic base, such as triethylamine (Et_3_N) [1,9,14,15,18,23] or *N*,*N*-diisopropylethylamine (DIPEA; iPr_2_NEt) [5,7,8,16,17,21]. These bases facilitate proton abstraction from the intermediate **4** (Scheme 1b). In cases where an additional base is not included in the reaction mixture [4,11], the amine nucleophile is generally used in excess, acting both as a Brønsted–Lowry base and a nucleophile. Alternatively, proton abstraction from intermediate **4** could be mediated by the lone electron pairs of solvent heteroatoms [2,5].

Overall, the versatility of the S_N_Ar regioselective reaction for C4 substitution is noteworthy, providing the corresponding 2-chloro-4-aminoquinazoline derivatives (**3**). In contrast, performing a second substitution at the C2 position, leading to the formation of the corresponding 2,4-diaminoquinazolines (Figure 1), typically requires more stringent conditions, such as higher temperatures (above 100 °C) [7,10,11,13,14,17], microwave irradiation [7,13,14], and/or a Buchwald−Hartwig amination reaction [10], likely due to the lower reactivity of the C2 position.

Despite the substantial experimental evidence, detailed chemical discussions on the molecular basis for the widely observed S_N_Ar regioselectivity favoring 4-position substitution in 2,4-dichloroquinazoline precursors are limited. This work presents in silico DFT calculations and 2D-NMR studies aiming to clarify the structural and electronic factors underlying the experimentally observed regioselectivity and to provide guidance for the structural confirmation of 4-amino-substituted product formation.

## 2. Results and Discussion

### 2.1. In Silico Studies and Mechanistic Insights

In order to understand, at a molecular level, the observed regioselectivity of S_N_Ar reactions starting from 2,4-dichloroquinazoline precursors, calculations were performed using a DFT hybrid generalized gradient approximation (GGA) functional ωB97X-D [29] with the 6-31G(d) basis set. The ωB97X-D functional includes dispersion corrections, which permits the modeling of intermolecular and intramolecular interactions, and the 6-31G(d) basis set includes polarization functions, enabling the representation of distorted electron clouds in the formation of transition states. The unsubstituted 2,4-dichoroquinazoline (**1a**; Table 2) and substituted 2,4-dichoroquinazolines (**1b**–**k**; Supplementary Material: Table S1) were used in our first analysis. After geometry optimization in the gas phase with ωB97X-D, a second geometry optimization was carried out using the conductor-like polarizable continuum model (C-PCM) for polar solvents available in Spartan’20. The inclusion of C-PCM refined the calculated properties, leading to a more accurate prediction of nucleophilic attack sites. As highlighted in Table 2, regarding unsubstituted 2,4-dichoroquinazoline (**1a**), the three different atomic charges (electrostatic, natural, and Mulliken) calculated on Spartan’20 indicate that carbon atoms at positions 2 (C2) and 4 (C4) are electron-deficient and that C2 is more electron-deficient than C4, as expected, since it is localized between two electron-withdrawing N atoms.

The most accepted mechanism for the typical S_N_Ar reaction is a simple two-step addition/elimination process, in which the intermediate Meisenheimer σ adduct is formed, although recent results are indicative that concerted and borderline mechanisms are also possible [30]. In any case, the nucleophile must approach the π-electron-rich system, so it would be initially expected a preference for the first step of the reaction to occur preferentially on C2; however, since in this step, the nucleophile must form a covalent bond with the substrate by means of electrons transfer to it, a strong frontier orbital influence is also expected in the S_N_Ar reaction mechanism. Atomic charges provide a measure of electron deficiency or surplus at a specific site, suggesting where the nucleophile might be attracted electrostatically. However, this factor alone may not account for the kinetic and orbital-driven aspects of the reaction mechanism. While atomic charges suggest an initial attraction, the reaction’s progress depends on the ability of the nucleophile to interact effectively with the molecular orbitals of the substrate.

In fact, we observed that C4 orbitals made a greater contribution to the LUMO (Table 2) of precursor **1a**, a result that could explain the observed regioselectivity of the replacement of the chlorine atom at position 4 of 2,4-dichloroquinazoline precursors by primary and secondary amine nucleophiles. It is important to note that the MO coefficients shown in Table 2 represent the two atomic orbitals of each carbon with the greatest contributions to LUMO. There was no significant variation between **1a** and substituted precursors **1b-1k** (Supplementary Material: Table S1) and, thus, **1a** was used as a model for the next calculations.

**Figure 2:**
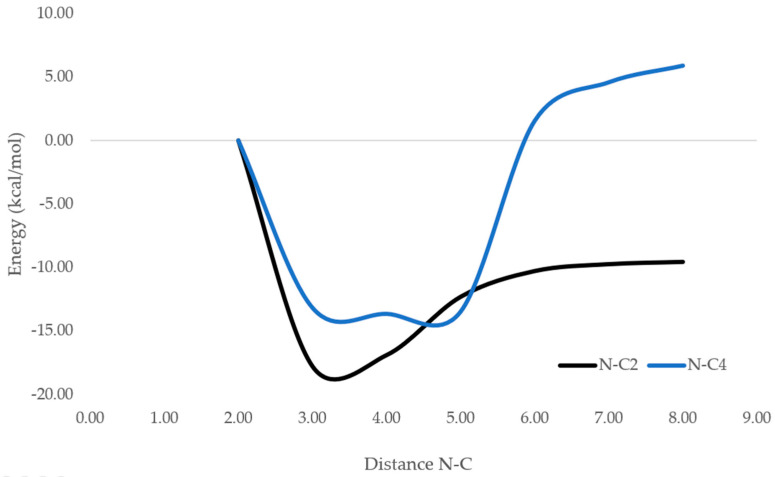
Potential energy surface analysis between (**2a**) and (**1a**) by varying the distance between the aniline N atom in relation to the C2 (black curve) and C4 (blue curve) atoms of the quinazoline moiety from 2 to 8 Å, with steps of 1 Å.

**Scheme 2:**
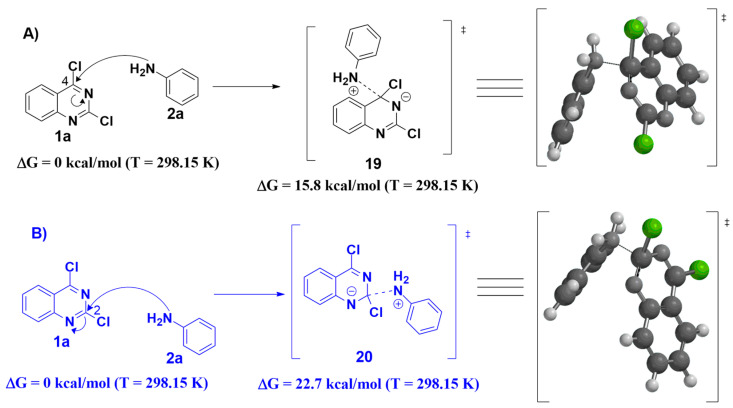
Activation energy determination for the nucleophilic aromatic substitution reaction at C4 (**A**) and C2 (**B**) of the 2,4-dichloro-quinazoline (**1a**) at the ωB97X-D/6-31G(d) level of theory using the C-PCM solvation model for polar solvents, available on Spartan’20. ^‡^ Transition state.

Since electrophilicity and LUMO coefficient are properties of the reactant molecule **1**, to establish a more adequate evaluation of the effect of the substitution center on the reaction mechanism, we modeled the nucleophilic attack of aniline (**2a**) to 2,4-dichloro-quinazoline (**1a**) at both positions and analyzed the transition states to obtain the corresponding activation energies, considering that lower activation energies favor faster reactions and influence regioselectivity (Scheme 2). Initially, we obtained a π-complex structure formed between (**2a**) and (**1a**) through a potential energy surface analysis between both systems by varying the distance between the aniline N atom in relation to the C2 and C4 atoms of the quinazoline moiety from 2 to 8 Å, with steps of 1 Å (Figure 2). From the lowest energy complex found, an equilibrium geometry calculation was performed. This step ensures that the complex is in its most stable configuration before proceeding to the transition state.

Next, the transition state geometry related to the nucleophilic attack of the aniline at C2 and C4 was calculated. As can be observed in Scheme 2, the transition state at C4 requires a lower activation energy, in accordance with the experimental results for the reaction regioselectivity. The transition state geometry was confirmed using vibrational analysis, which allows for the verification of the reaction pathway. In this analysis, one imaginary frequency was identified for each transition state, which corresponds to the reaction coordinate. For the two transition states, the imaginary frequencies were found to be i47 (**19**) and i255 (**20**), providing evidence that the calculated transition states align with the expected reaction mechanism.

### 2.2. Synthesis and S_N_Ar Regioselectivity Confirmation by 2D-NMR Studies

Our research group has been investigating the C4 regioselective S_N_Ar reaction for the synthesis of bioactive 4-aminoquinazolines. To validate the obtained theoretical data, we examined our internal chemical repository, the LASSBio Chemical Library [31], to identify relevant quinazoline systems for more detailed analysis. This approach aimed not only to investigate the reaction regioselectivity but also to provide better guidance for the structural confirmation of 4-amino-substituted product formation.

**Scheme 3:**
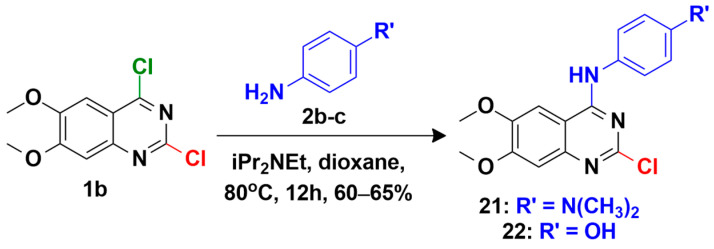
Regioselective S_N_Ar in the synthesis of 2-chloro-4-anilinoquinazolines **21** and **22** [5].

A previous report from our research group described the application of the 4-position regioselective S_N_Ar in the synthesis of a congener series of bioactive 2-chloro-4-anilinoquinazolines (Supplementary Material: Table S2) as multi-target directed ligands (MTDLs) designed to inhibit epidermal growth factor receptor (EGFR) and vascular endothelial growth factor receptor (VEGFR) tyrosine kinases for cancer treatment [5]. Herein, we performed the synthesis of 2-chloro-4-anilinoquinazolines **21** and **22** according to our described methodology [5] and performed 2D-NMR studies for unambiguous confirmation of the reaction’s regioselective substitution of the chlorine atom in position 4 of the 6,7-dimethoxy-2,4-dichloroquinazoline synthetic precursor **1b** (Scheme 3), demonstrating that 2D-NMR analyses are suitable and adequate to confirm the obtained C4 substitution pattern.

These results will be useful to support subsequent studies, since, in most synthetic reports, it is assumed that the reaction occurred in a regioselective manner, as expected, but experimental confirmation of regioselectivity is often not described along with the synthetic data.

Initially, a two-dimensional nuclear magnetic resonance (2D-NMR) NOESY experiment was carried out. The 2D-NOESY technique is based on the detection of the NOE (nuclear Overhauser effect) between ^1^H nuclei close in space, manifested as a cross-peak between the two ^1^H NMR resonance spectra.

This experiment can be easily run to confirm the 4-position quinazoline ring substitution pattern in the synthesis of 2-chloro-4-aminoquinazoline products from 2,4-dichloroquinazolines when a primary amine is employed as a nucleophile, as illustrated in Scheme 3 for antitumor prototypes **21** and **22**, respectively. These experimental data provide valuable support for regioselectivity confirmation when 2-position or 4-position regioisomers are possible.

For this purpose, 4-aminoquinazolines **21** and **22** were initially analyzed using ^1^H NMR in DMSO-*d_6_* solution and expected correlations for protons nearby in space were anticipated.

**Figure 3:**
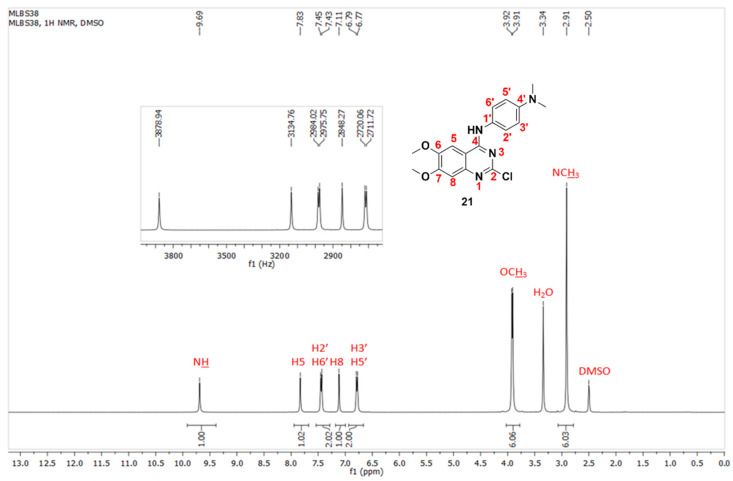
^1^H NMR (800 MHz; 25 °C; DMSO-*d_6_*) spectrum and signal assignment for bioactive 2-chloro-4-anilinoquinazoline **21** (LASSBio-1812).

**Figure 4:**
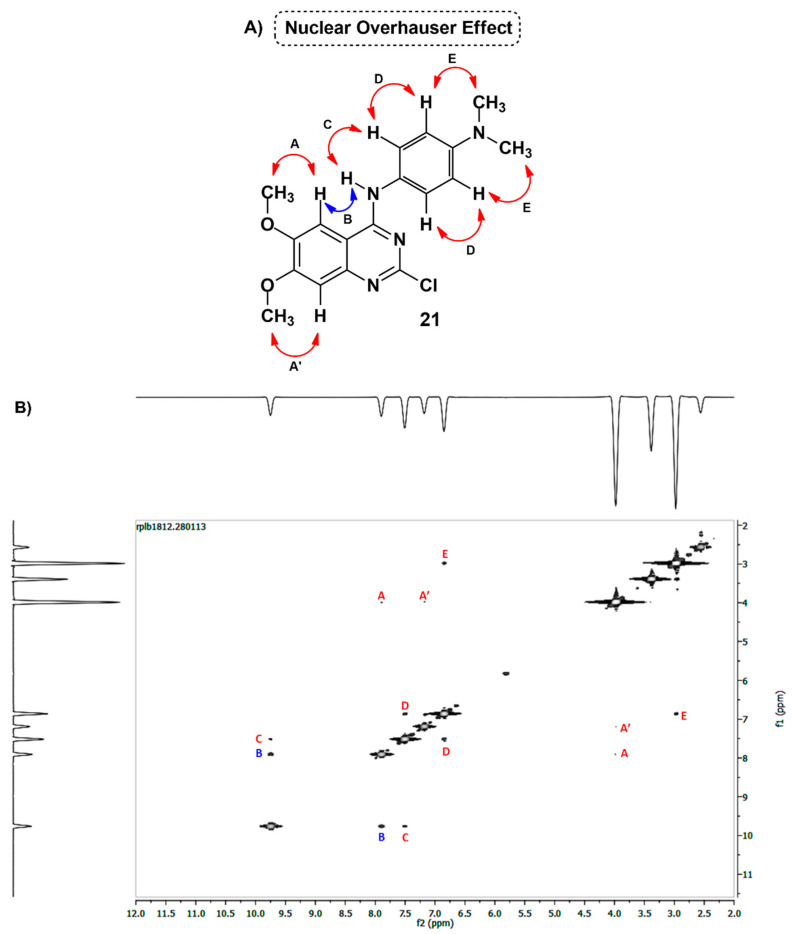
2D-NOESY predicted (**A**) and observed (**B**) (800 MHz; 25 °C; DMSO-*d_6_*) correlations for bioactive 2-chloro-4-anilinoquinazoline **21** (LASSBio-1812). The observed NOE correlations are indicated in red, with the spatial proximity between the NH and H5 signals confirmed as correlation B, highlighted in blue.

For 4-aminoquinazoline **21**, after ^1^H NMR signals’ assignment based on chemical shifts, coupling constants and integration, as indicated in Figure 3, all predicted NOE correlations were clearly observed in the 2D-NOESY analysis (Figure 4), highlighting the confirmed space proximity between the NH signal at δ 9.69 ppm and the quinazoline core H5 signal at δ 7.83 ppm, represented in blue as correlation B.

**Figure 5:**
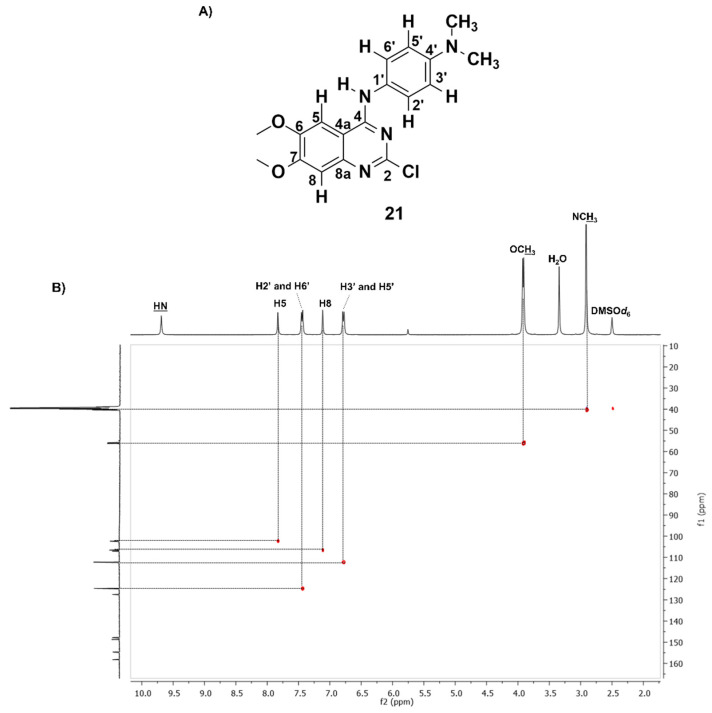
Chemical structure (**A**) and 2D-HSQC NMR spectrum (**B**) of 2-chloro-4-anilinoquinazoline **21** (LASSBio-1812) (500 MHz spectrometer; 25 °C; DMSO-*d_6_*). Hydrogen and carbon signals are assigned according to the chemical structure atom numbers.

**Figure 6:**
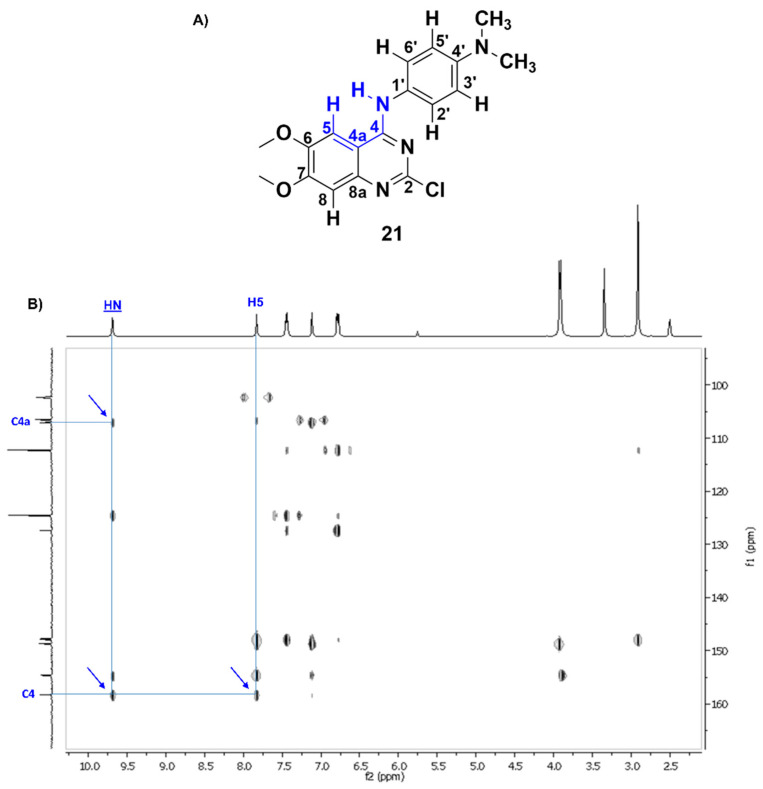
Chemical structure (**A**) and 2D-HMBC NMR spectrum (**B**) of 2-chloro-4-anilinoquinazoline **21** (LASSBio-1812) (500 MHz spectrometer; 25 °C; DMSO-*d_6_*). The annotated cross-peaks indicate the ^3^*J*_CH_ coupling between C4a and the NH hydrogen and the ^3^*J*_CH_ and ^2^*J*_CH_ coupling of C4 with hydrogens H5 and NH, respectively, as highlighted in blue in the chemical structure.

Further, hydrogen–carbon correlations allowed for the signals’ unambiguous assignment to the ^1^H and ^13^C NMR spectra of **21** through 2D-HSQC (heteronuclear single quantum coherence, Figure 5) and 2D-HMBC (heteronuclear multiple bond correlation, Figure 6) analysis, aiming at ensuring the reliability of the performed signals’ assignment.

To determine the hydrogen and carbon single-bond correlations, a 2D-HSQC experiment was executed. The 2D-HSQC spectrum confirmed the correlation between the aromatic hydrogens and the corresponding carbons, as expected, e.g., hydrogen-H5 (7.83 ppm) and carbon-C5 (102.3 ppm), hydrogen-H8 (7.11 ppm) and carbon-C8 (106.6 ppm), hydrogens-H3′ and H5′ (6.78 ppm) with carbons-C3′ and C5′ (112.3 ppm), and hydrogens-H2′ and H6′ (7.44) and carbons-C2′ and C6′ (124.6) (Figure 5).

Additionally, the 2D-HMBC experiment provided the unequivocal assignment of molecules’ quaternary carbons (Figure 6 and Supplementary Material—Figures S1–S4) and allowed, once again, for the confirmation of the expected 4-substitution pattern of 4-anilinoquinazoline **21**, as was already well determined from the NOESY spectrum (Figure 4).

The cross-peaks highlighted in Figure 6 indicate the ^3^*J*_CH_ coupling between C4a and the NH hydrogen and the ^3^*J*_CH_ and ^2^*J*_CH_ coupling of C4 with hydrogens H5 and NH, respectively.

**Figure 7:**
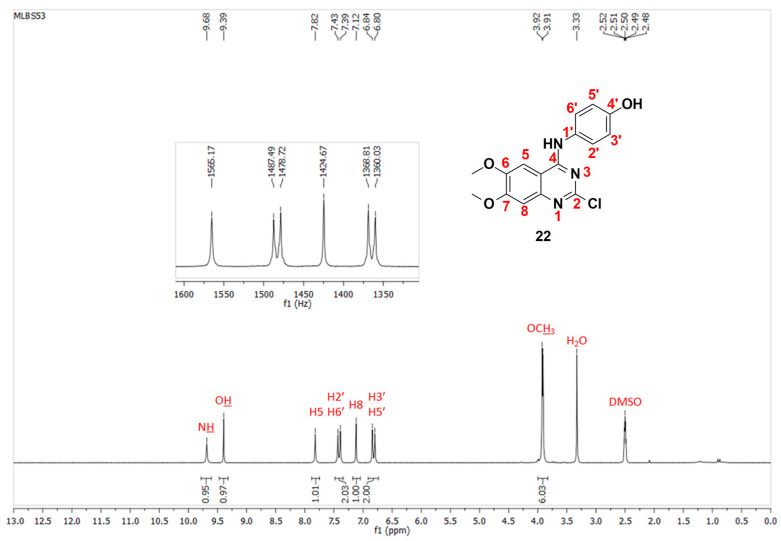
^1^H NMR (800 MHz; 25 °C; DMSO-*d_6_*) spectrum and signal assignment for bioactive 2-chloro-4-anilinoquinazoline **22** (LASSBio-1821).

**Figure 8:**
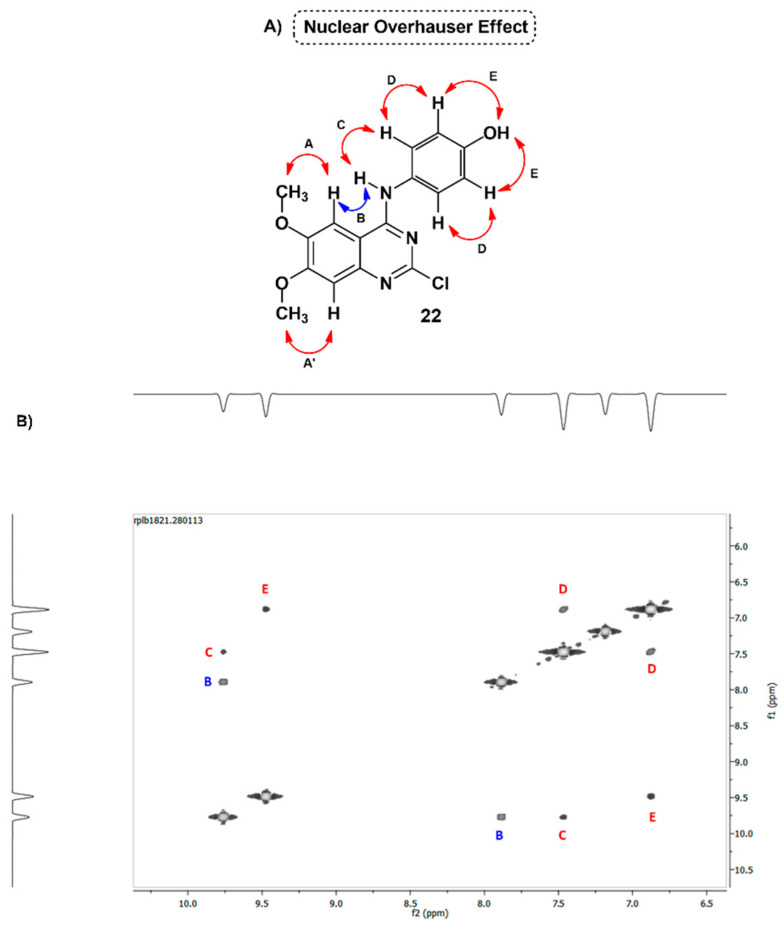
2D-NOESY predicted (**A**) and observed (**B**) (800 MHz; 25 °C; DMSO-*d_6_*; chemical shift ranges from 6.5 ppm to 11.0 ppm) correlations for bioactive 2-chloro-4-anilinoquinazoline **22** (LASSBio-1821). The observed NOE correlations are indicated in red, with the spatial proximity between the NH and H5 signals confirmed as correlation B, highlighted in blue.

Regarding the structural analog **22** (LASSBio-1821), similar results were obtained for ^1^H NMR (Figure 7) and 2D-NOESY spectra (Figure 8). In the 2D-NOESY analysis of **22**, most of the anticipated NOE correlations were detectable, highlighting, once again in blue, correlation B demonstrating the space proximity between the NH signal at δ 9.68 ppm and the quinazoline core H5 signal at δ 7.82 ppm.

In fact, experimental confirmation of regioselectivity should be considered as recommended when more than one regioisomer represents a reasonable product for the described reaction. On the other hand, it is highly expected that the regioselectivity for the substitution at position 4 of quinazoline nucleus is preserved when a S_N_Ar reaction is performed starting from 2,4-dichloro-quinazoline precursors and anilines [2,3,5,10], benzylamines [6,14,23], and/or aliphatic primary or secondary amines [1,2,4,6,7,8,9,10,11], as demonstrated by a set of reports depicted in Table 1.

## 3. Materials and Methods

### 3.1. Synthesis and NMR Studies

2-chloro-4-anilinoquinazolines **21** and **22** were obtained according to our previously reported methodology (Scheme 3) [5]. A mixture of 0.60 mmol of 6,7-dimethoxy-2,4-dichloroquinazoline synthetic precursor **1b**, 0.60 mmol of the corresponding aniline and 2.18 mmol *N*,*N*-diisopropylethylamine (DIPEA; iPr_2_NEt) in dioxane (6 mL) was stirred at 80 °C for 12 h under an inert atmosphere. The mixture was cooled to room temperature after reactant consumption, and the reaction mixture was diluted with water and extracted with ethyl acetate. The extracts were combined and dried over anhydrous Na_2_SO_4_. The solvent was evaporated, and the residue was purified as described.

^1^H nuclear magnetic resonance (NMR) and 2D-NOESY spectra were determined in 10 mg/0.6 mL DMSO-*d_6_* solutions using a Bruker UltraStabilized 800 spectrometer (CNRMN-UFRJ, Rio de Janeiro, RJ, Brazil). ^13^C nuclear magnetic resonance (NMR) spectra were determined in 20 mg/0.6 mL DMSO-*d_6_* solutions using a Bruker Avance 400 spectrometer. 2D-HSQC and HMBC spectra were determined in 10 mg/0.6 mL DMSO-*d_6_* solutions using a Varian 500 spectrometer (LAMAR-IPPN-UFRJ, Rio de Janeiro, RJ, Brazil).

The chemical shifts are given in parts per million (δ) from solvent residual peaks and the coupling constant values (*J*) are given in Hz. Signal multiplicities are represented by s (singlet) and d (doublet).

#### 3.1.1. *N*^1^-(2-Chloro-6,7-dimethoxyquinazolin-4-yl)-*N*^4^,*N*^4^-dimethylbenzene-1,4-diamine (**21**; LASSBio-1812)

2-chloro-4-anilinoquinazoline **21** was obtained as a yellow powder at a 65% yield via condensation of **1b** with 4-(*N*,*N*-dimethylamino)-aniline (**2b**) and further purification by flash chromatography (silica gel; dichloromethane:methanol, 99:1), mp. 171–173 °C. NMR data obtained for compound **21** (Figure 4) are consistent with previous reports [5]. ^1^H NMR (800 MHz, DMSO-*d_6_*) δ (ppm): 2.91 (s, 6H, RN(CH_3_)_2_); 3.91 (s, 3H, OCH_3_); 3.92 (s, 3H, OCH_3_); 6.78 (d, 2H, J = 8.3 Hz, H3′ and H5′); 7.11 (s, 1H, H8); 7.44 (d, 2H, J = 8.3 Hz, H2′ and H6′); 7.83 (s, 1H, H5); 9.69 (s, 1H, NH). ^13^C NMR (200 MHz, DMSO-*d_6_*) δ (ppm): 40.3 (ArNCH_3_)_2_); 55.9 and 56.2 (ArOCH_3_); 102.3 (C5); 106.6 (C8); 107.1 (C4a); 112.3 (C3′ and C5′); 124.6 (C2′ and C6′); 127.4 (C1′); 147.8 (C4′); 147.9 (C8a); 148.7 (C6); 154.6 (C7); 154.8 (C2); 158.3 (C4).

#### 3.1.2. 4-((2-Chloro-6,7-dimethoxyquinazolin-4-yl)amino)phenol (**22**; LASSBio-1821)

2-chloro-4-anilinoquinazoline **22** was obtained as a beige powder at a 60% yield via condensation of **1b** with 4-aminofenol (**2c**) and further purification by flash chromatography (silica gel; dichloromethane:methanol, 99:1), mp. > 300 °C. NMR data obtained for compound **22** (Figure 8) are consistent with previous reports [5]. ^1^H NMR (500 MHz, DMSO-*d_6_*) δ (ppm): 3.91 (s, 3H, OCH_3_); 3.92 (s, 3H, OCH_3_); 6.82 (d, 2H, J = 8.8 Hz, H3′ and H5′); 7.12 (s, 1H, H8); 7.41 (d, 2H, J = 8.8 Hz, H2′ and H6′); 7.82 (s, 1H, H5); 9.39 (s, 1H, OH); 9.68 (s, 1H, NH). ^13^C NMR (125 MHz, DMSO-*d_6_*) δ (ppm): 55.8 and 56.2 (ArOCH_3_); 102.3 (C5); 106.6 (C8); 107.0 (C4a); 115.1 (C3′ and C5′); 125.1 (C2′ and C6′); 129.6 (C1′); 147.8 (C8a); 148.8 (C6); 154.5 (C7); 154.6 (C4′); 154.7 (C2); 158.3 (C4).

### 3.2. In Silico Studies

All geometry optimization and transition state calculations were performed using the ωB97X-D/6-31G(d) level of theory with the C-PCM solvation model for polar solvents (dielectric constant of 37), available in Spartan’20. The activation energy of the modeled reactions started with the π-complexes formed by the 2,4-dichloro-quinazoline (**1a**) and aniline (**2a**). The π-complexes were determined through potential energy surface scan analyses varying the distance formed between the nitrogen atom of the aniline (**2a**) in relation to the carbon atoms in positions 2 and 4 of the quinazoline (**1a**). Thus, 2 complexes were created, varying the distance in the range of 2 to 8 Å with steps of 1 Å. From the lowest energy complexes, geometry optimization calculations were performed using ωB97X-D/6-31G(d). The resulting geometries were used as initial reference structures for activation energy determination. Transition state geometry calculations were performed considering the additional step of the S_N_Ar mechanism as the slowest step. This step was modeled using ωB97X-D/6-31G(d), and the vibrational analysis was implemented to confirm the observed transition states.

## 4. Conclusions

The 4-aminoquinazoline framework is characterized as a privileged structure for the design of novel drug candidates due to its pleiotropic pharmacological profile. In this context, the regioselective nucleophilic aromatic substitution (S_N_Ar) for replacement of the chlorine atom at position 4 of 2,4-dichloroquinazoline precursors is extensively employed in the synthesis of novel bioactive 2-chloro-4-aminoquinazolines and/or 2,4-diaminoquinazolines. Several previous reports have demonstrated that 4-position regioselectivity is preserved within different S_N_Ar reaction conditions with primary or secondary amines.

The DFT calculations reported herein help to explain the widely observed regioselectivity, as the carbon atom at position 4 of the heterocyclic system has a greater LUMO coefficient and, accordingly, the calculated activation energy associated with the nucleophilic attack for the first step of the S_N_Ar is lower when the nucleophile approaches the 2,4-dichoroquinazoline precursor at this position.

Finally, taking into account that the experimental confirmation of regioselectivity should be considered as recommended when more than one regioisomer represent a reasonable product for the performed reaction, integration of 2D-NMR methods can be considered as suitable for structural characterization aiming at the confirmation of 4-position quinazoline ring substitution pattern.

## Figures and Tables

**Table 1:** Regioselective S_N_Ar reaction conditions described for conversion of 2,4-dichloro-quinazoline precursors (**1a**–**k**) into 2-chloro-4-aminoquinazolines. The original reference for each reaction condition is provided in square brackets.

2,4-Dichloro-quinazoline Precursor	Nucleophile	Organic or Inorganic Base	Solvent	Reaction Temperature	Reaction Time (According to Reaction Conditions)
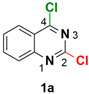	[1,4,6,7,10,16,23,24]: Aliphatic and benzylic amines; [3,5,10]: Anilines; [20]: Benzylic amines.	[1,15,23]: Et_3_N; [3,6,10,24]: NaOAc; [5,7,16]: iPr_2_NEt.	[1,4,15,23]: THF; [3,6,10,24]: THF/H_2_O; [5,16,20]: Ethanol; [5]: Dioxane or 2-propanol; [7]: Acetonitrile.	Lower: [1,4,9,15,19]: r.t.; Higher: [5,8,16]: ~80–82 °C.	Faster: [4]: Aliphatic amines, THF, r.t., 0.5–3 h; Slower: [5]: Anilines, ethanol or 2-propanol, reflux, 24 h.
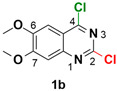	[2,7,11]: Aliphatic amines; [2,5,11] Anilines; [7,11]: Benzylic amines.	[5,7]: iPr_2_NEt.	[2]: THF; [2,5]: Ethanol or 2-propanol; [5]: Dioxane; [7,11]: Acetonitrile.	Lower: [2,7]: r.t.; Higher: [2,5]: ~80–82 °C.	Faster: [2]: Anilines, ethanol or 2-propanol, reflux, 2 h; Slower: [2]: Aliphatic amines, THF, r.t., 24 h.
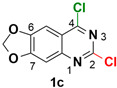	[5] Anilines.	[5]: iPr_2_NEt.	[5]: Dioxane, ethanol or 2-propanol.	[5]: ~80–82 °C.	Faster: [5]: Anilines, iPr_2_NEt, dioxane, 80 °C, 12 h; Slower: [5]: Anilines, ethanol or 2-propanol, reflux, 24 h.
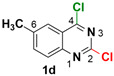	[9]: Aliphatic amines.	[9]: Et_3_N.	[9]: Methanol.	[9]: r.t.	[9]: Aliphatic amines, Et_3_N, methanol, r.t., ~12 h.
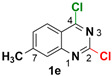	[14]: Benzylic amines.	[14]: Et_3_N.	[14]: CH_2_Cl_2._	[14]: r.t.	[9]: Benzylic amines, Et_3_N, CH_2_Cl_2_, r.t., ~12 h.
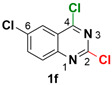	[8,13]: Aliphatic amines; [21]: Anilines.	[8,21]: iPr_2_NEt.	[8,13]: Ethanol; [21]: DMF.	[21]: 0 °C; [13]: r.t.; [8]: 78 °C.	Faster: [9]: Aliphatic amines, ethanol, r.t., ~1 h. Slower: [21]: Anilines, iPr_2_NEt, DMF, 0 °C, 4 h.
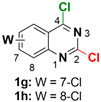	[8]: Aliphatic amines.	[8]: iPr_2_NEt.	[8]: Ethanol.	[8]: 78 °C.	[8]: Aliphatic amines, iPr_2_NEt, ethanol, 78 °C, 3–4 h.
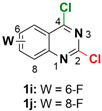	[20]: Benzylic amines.	[20]: None.	[20]: Ethanol.	[20]: 78 °C.	Reaction time not informed.
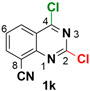	[17]: Benzylic amines.	[17]: iPr_2_NEt.	[17]: THF.	[17]: r.t.	[17]: Benzylic amines, iPr_2_NEt, THF, r.t., 16 h.

**Table 2:** Atomic charges and LUMO coefficients calculated for C2 and C4 atoms of 2,4-dichloro-quinazoline precursor **1a** with the ωB97X-D/6-31G(d) level of theory using the C-PCM solvation model for polar solvents.

		Atomic Charges				
Compound	Atom	Electrostatic	Mulliken	Natural	6-31G(d) Split Valence LUMO Coefficients	ωB97X-D/6-31G(d) LUMO
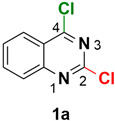	C4	0.467	0.086	0.292	3p_y_ = 0.37041 3p_y_’ = 0.39117	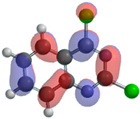
	C2	0.718	0.249	0.410	3p_y_ = −0.16307 3p_y_’ = −0.15101	

## Data Availability

The original data presented in the study are included in the article and Supplementary Material; further inquiries are available on request from the corresponding author.

## Supplementary Materials

The following supporting information can be downloaded at: https://www.mdpi.com/article/10.3390/molecules29246021/s1, Table S1: Atomic charges and LUMO coefficients calculated for C2 and C4 atoms of 2,4-dichloro-quinazoline precursors **1b**–**k** with the ωB97X-D/6-31G(d) level of theory using the C-PCM solvation model for polar solvents; Table S2: Synthetic methodology, reaction yields and chemical shifts (δ; ppm) of the representative signals of the ^1^H NMR spectra (400 MHz; 25 °C; DMSO-*d*_6_) of previously synthesized 2-chloro-4-aniline-quinazoline derivatives **21**, **22**, **23a**–**g**; Figure S1: 2D-HMBC NMR spectrum for LASSBio-1812 (**21**). The highlighted rectangles are zoomed in Figures S2–S4 to improve visualization and clarity; Figure S2. Part A of the 2D HMBC NMR spectra of LASSBio-1812 (**21**). C8 (red) showed a cross-peak with H5 (red line) and C4a (green) showed cross-peaks with NH and H8 (green line); Figure S3. Part B of 2D HMBC NMR of LASSBio-1812 (**21**). C4′ (red) showed cross-peaks with H2′, H6′ and NCH_3_ (red line), and C8a (green) showed a cross-peaks with H5 (green line), and C6 (black) showed cross-peaks with H8 and OCH_3_ (black line); Figure S4. Part C of 2D HMBC NMR of LASSBio-1812 (**21**). C7 (red) showed cross-peaks with H5 and OCH_3_ (red line), C2 (green) showed a cross-peak with NH (green line), and C4 (black) showed cross-peaks with NH, H5 and H8 (black line).
