# Supplementary material for: Regioselective Nucleophilic Aromatic Substitution: Theoretical and Experimental Insights into 4-Aminoquinazoline Synthesis as a Privileged Structure in Medicinal Chemistry
Source: Molecules. 2024 Dec 20;29(24):6021. doi: 10.3390/molecules29246021 (PMC11678402; doi:10.3390/molecules29246021)
Supplement: Supplementary file 1 [file molecules-29-06021-s001.zip › molecules-3348724-supplementary.pdf]

Regioselective Nucleophilic Aromatic Substitution: Theoretical and Experimental Insights into 4-Aminoquinazoline Synthesis as a Privileged Structure in Medicinal Chemistry

Maria Letícia de Castro Barbosa [1,2,*], Pedro de Sena Murteira Pinheiro [2,3], Raissa Alves da Conceição [4], José Ricardo Pires [5], Lucas Silva Franco [2,3], Carlos Mauricio R. Sant'Anna [2,3,6], Eliezer J. Barreiro [2,3] and Lídia Moreira Lima [2,3,*]

1 Faculty of Pharmacy, Department of Pharmaceutical Sciences, Federal University of Juiz de Fora, Juiz de Fora 36036-900, MG, Brazil
2 Laboratory of Evaluation and Synthesis of Bioactive Substances (LASSBio), Institute of Biomedical Sciences, Federal University of Rio de Janeiro, Rio de Janeiro 21941-902, RJ, Brazil; pedro.pinheiro@icb.ufrj.br (P.d.S.M.P.); silvafrancolucas@gmail.com (L.S.F.); santanna@ufrrj.br (C.M.R.S.); ejbarreiro@ccsdecania.ufrj.br (E.J.B.)
3 National Institute of Science and Technology in Drugs and Medicines (INCT-INOFAR), Rio de Janeiro 21941-902, RJ, Brazil
4 Faculty of Pharmacy, Federal University of Rio de Janeiro, Rio de Janeiro 21941-902, RJ, Brazil; raissa.conceicao9@gmail.com
5 Institute of Medical Biochemistry Leopoldo de Meis (IBqM), Federal University of Rio de Janeiro, Rio de Janeiro 21941-902, RJ, Brazil; murari@bioqmed.ufrj.br
6 Institute of Chemistry, Federal Rural University of Rio de Janeiro, Seropédica 23970-000, RJ, Brazil
* Correspondence: marialeticia.barbosa@ufjf.br (M.L.d.C.B.); lidialima@ufrj.br (L.M.L.)

I. Atomic charges and LUMO coefficient calculations for substituted 2,4-dichloroquinazolines (1b-k)

Table S1: Atomic charges and LUMO coefficients calculated for C2 and C4 atoms of 2,4-dichloroquinazoline precursors **1b-k** with the ωB97X-D/6-31G(d) level of theory using the C-PCM solvation model for polar solvents.

		Atomic charges				
Compound	Atom	Electrostatic	Mulliken	Natural	6-31G(d) split valence LUMO coefficients	ωB97X-D/6-31G(d) LUMO
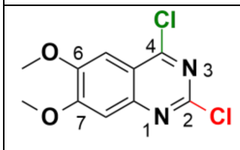 **1b**	C4	0.521	0.071	0.283	$3p_x$ = 0.36167<br>$3p_y'$ = 0.38444	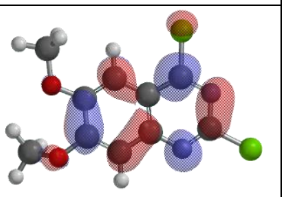
	C2	0.700	0.245	0.405	$3p_y$ = -0.14075<br>$3p_y'$ = -0.13137	
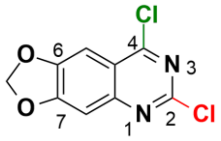 **1c**	C4	0.529	0.067	0.272	$3p_x$ = 0.36751<br>$3p_y'$ = 0.39381	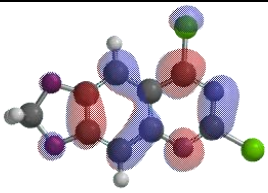
	C2	0.703	0.245	0.403	$3p_y$ = -0.14784<br>$3p_y'$ = -0.14017	
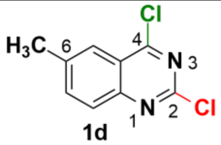 **1d**	C4	0.514	0.082	0.285	$3p_y$ = 0.36642<br>$3p_y'$ = 0.39136	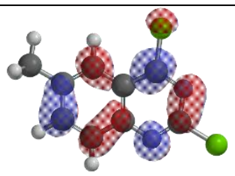
	C2	0.724	0.246	0.403	$3p_y$ = -0.15104<br>$3p_y'$ = -0.14145	
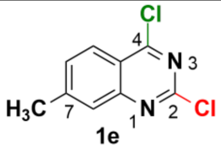 **1e**	C4	0.521	0.083	0.286	$3p_y$ = 0.36937<br>$3p_y'$ = 0.39452	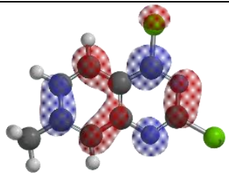
	C2	0.719	.0248	0.406	$3p_y$ = -0.16350<br>$3p_y'$ = -0.15440	

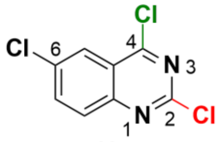 1f	C4	0.486	0.092	0.287	$3p_y$ = 0.36294<br>$3p_y'$ = 0.38207	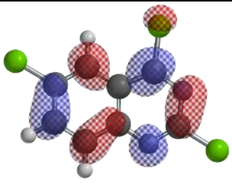
	C2	0.729	0.252	0.407	$3p_y$ = -0.16071<br>$3p_y'$ = -0.14746	
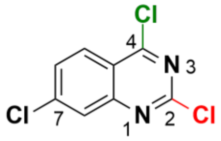 1g	C4	0.502	0.091	0.289	$3p_y$ = 0.36194<br>$3p_y'$ = 0.38136	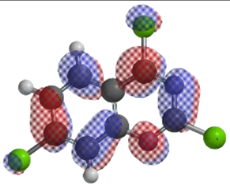
	C2	0.728	0.253	0.410	$3p_y$ = -0.15977<br>$3p_y'$ = -0.14627	
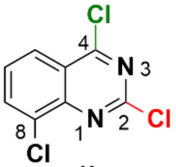 1h	C4	0.497	0.089	0.290	$3p_y$ = 0.36359<br>$3p_y'$ = 0.38312	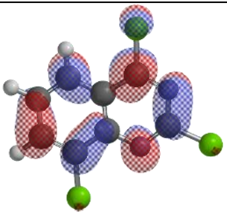
	C2	0.740	0.254	0.412	$3p_y$ = -0.17041<br>$3p_y'$ = -0.15787	
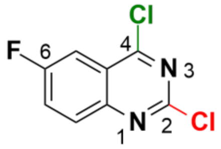 1i	C4	0.506	0.087	0.283	$3p_y$ = 0.36018<br>$3p_y'$ = 0.38157	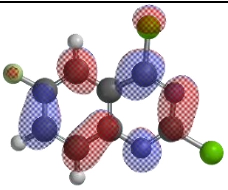
	C2	0.726	0.248	0.402	$3p_y$ = -0.14023<br>$3p_y'$ = -0.12790	
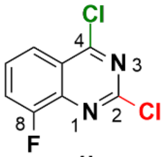 1j	C4	0.487	0.087	0.288	$3p_y$ = 0.37590<br>$3p_y'$ = 0.39928	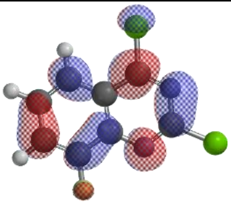
	C2	0.723	0.254	0.407	$3p_y$ = -0.15840<br>$3p_y'$ = -0.14980	
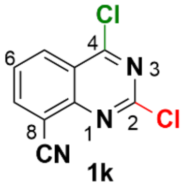 1k	C4	0.495	0.093	0.292	$3p_y$ = 0.32365<br>$3p_y'$ = 0.33171	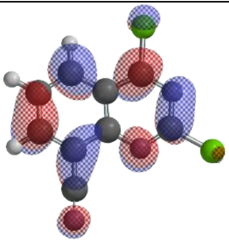
	C2	0.736	0.255	0.416	$3p_y$ = -0.18272<br>$3p_y'$ = -0.16344	

II. Bioactive 4-aminoquinazolines designed as multi-target directed ligands (MTDLs) and synthesized through the C-4 regioselective SNAr reaction in our previous studies

Table S2: Synthetic methodology, reaction yields and chemical shifts (δ; ppm) of the representative signals of the ^{1}H NMR spectra (400 MHz; 25°C; DMSO-*d6*) of previously synthesized 2-chloro-4-aniline-quinazoline derivatives **21, 22, 23a-g** [1].

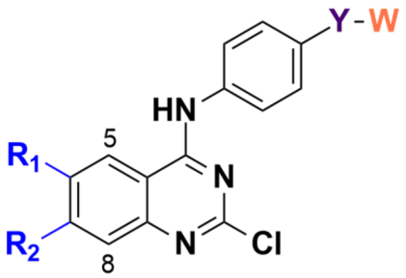

21, 22, 23a-g

Compound	R_1, R_2	Y	W	Methodology	Reaction Yield	H8	H5	NH
**21**	OCH_3, OCH_3	-	$N(CH_3)_2$	A: iPr_2NEt, dioxane, 80 °C, 12h.	65%	7,11 ppm (s)	7,83 ppm (s)	9,69 ppm (s)
**22**	OCH_3, OCH_3	-	OH	A: iPr_2NEt, dioxane, 80 °C, 12h.	60%	7,12 ppm (s)	7,82 ppm (s)	9,68 ppm (s)
**23a**	OCH_2O	-	$N(CH_3)_2$	A: iPr_2NEt, dioxane, 80 °C, 12h.	66%	7,09 ppm (s)	7,92 ppm (s)	9,55 ppm (s)
**23b**	OCH_3, OCH_3	SO_2	NH_2	B: Ethanol or 2-propanol, reflux, 24h.	68%	7,19 ppm (s)	7,95 ppm (m)	10,13 ppm (s)
**23c**	OCH_2O	SO_2	NH_2	B: Ethanol or 2-propanol, reflux, 24h.	67%	7,18 ppm (s)	7,99 ppm (s)	9,93 ppm (s)
**23d**	OCH_3, OCH_3	SO_2	$NHCH_3$	B: Ethanol or 2-propanol, reflux, 24h.	68%	7,20 ppm (s)	7,90 ppm (s)	10,08 ppm (s)
**23e**	OCH_3, OCH_3	SO_2	OH	B: Ethanol or 2-propanol, reflux, 24h.	66%	7,18 ppm (s)	7,90 ppm (s)	9,92 ppm (s)
**23f**	OCH_3, OCH_3	C=O	OH	B: Ethanol or 2-propanol, reflux, 24h.	73%	7,18 ppm (s)	8,11 ppm (s)	10,34 ppm (s)
**23g**	OCH_3, OCH_3	C=O	NH_2	B: Ethanol or 2-propanol, reflux, 24h.	69%	7,18 ppm (s)	8,03 ppm (s)	10,26 ppm (s)

III. Detailed signal assignment for 2D-HMBC NMR spectrum of LASSBio-1812 (21)

The 2D-HMBC NMR experiment indicates carbon-hydrogen correlations between nuclei separated by multiple bonds, usually two and three bonds. In some cases, it is also possible to observe the correlations between nuclei separated by four bonds. This spectrum is, therefore, useful for unambiguous assignment of molecules' quaternary carbons.

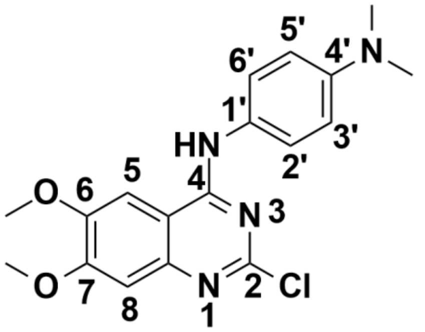

LASSBio-1812
(21)

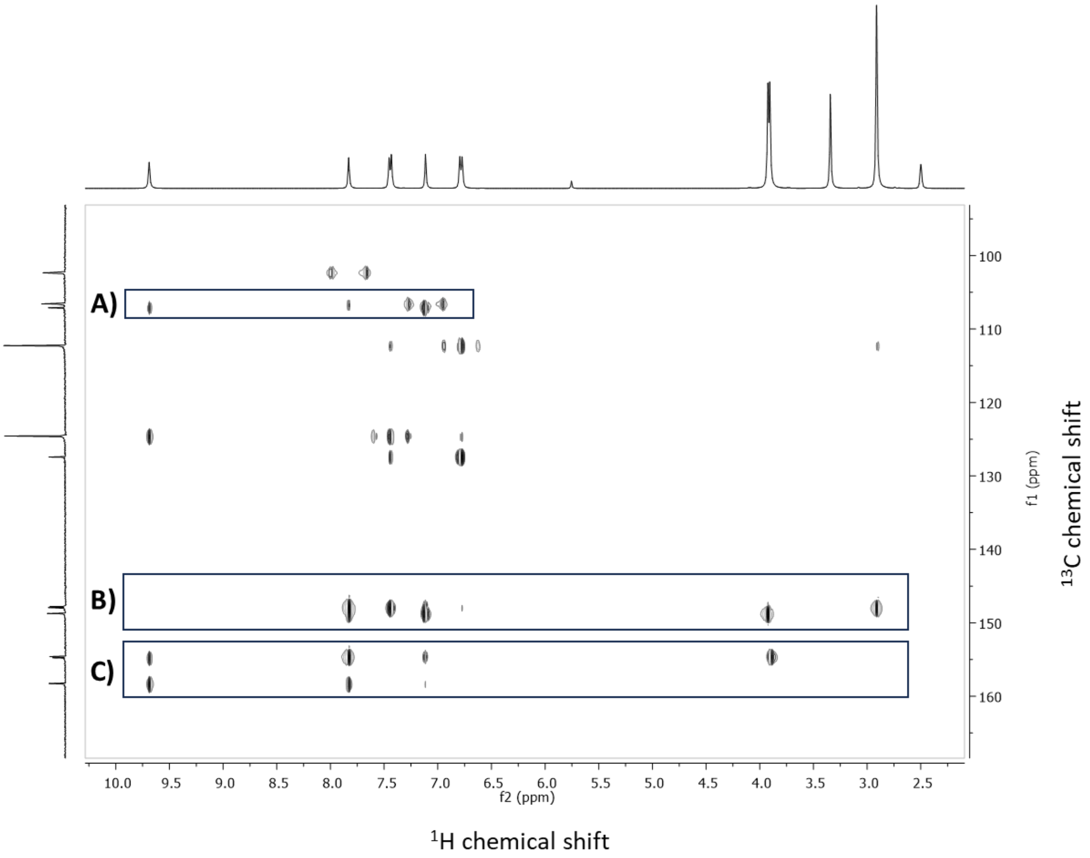

Figure S1. 2D-HMBC NMR spectrum for LASSBio-1812 (**21**). The highlighted rectangles are zoomed in Figures S2-S4 to improve visualization and clarity.

The chemical shift at 107.1 ppm was attributed to C4a due to cross-peaks that indicated $^{3}J_{CH}$ coupling with hydrogens NH and H8 (Figure S2).

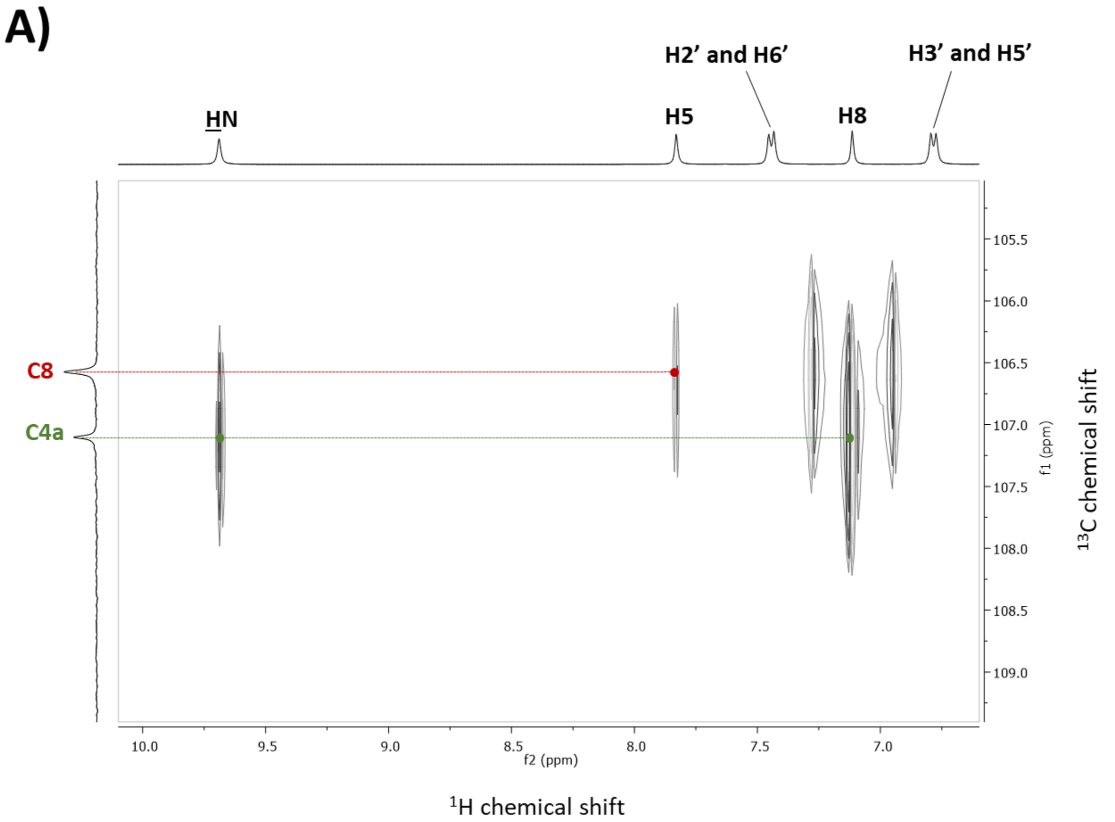

Figure S2. Part A of the 2D HMBC NMR spectra of LASSBio-1812 (**21**). C8 (red) showed a cross-peak with H5 (red line) and C4a (green) showed cross-peaks with NH and H8 (green line).

The cross-peaks with H3′ and H5′ and H2′ and H6′ indicated the ${}^3J_{CH}$ and ${}^2J_{CH}$ coupling, respectively, with C1′ at 127.4 ppm (Figure S1). The chemical shift at 147.8 ppm was attributed to C4′ due to cross-peaks with H2′, H6′ and $(ArNCH_3)_2$. This peak could be differentiated of C8a at 147.9 ppm, which presented a cross-peak with H5. The cross-peaks with hydrogens H8 and OCH_3 indicated the ${}^3J_{CH}$ to C6 at 148.7 ppm (Figure S3).

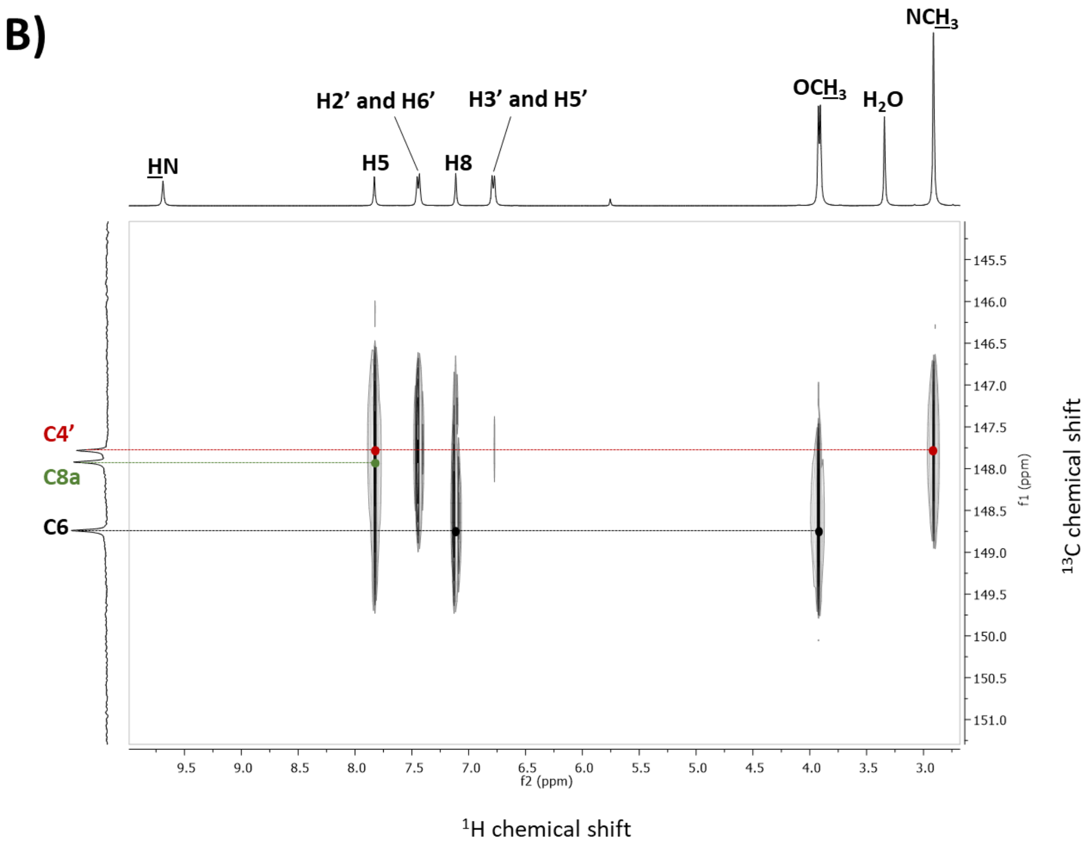

Figure S3. Part B of 2D HMBC NMR of LASSBio-1812 (**21**). C4′ (red) showed cross-peaks with H2′, H6′ and NCH_3 (red line), and C8a (green) showed a cross-peaks with H5 (green line), and C6 (black) showed cross-peaks with H8 and OCH_3 (black line).

The C7 showed the chemical shift at 154.6 ppm confirmed by the cross-peaks with H5 and OCH_3 by $^3J_{CH}$ coupling and it was also possible to observe the cross-peak with H8 indicating the $^2J_{CH}$ coupling. The chemical shift at 154.8 ppm confirmed C2 due to the only $^4J_{CH}$ coupling with NH (9.69 ppm). Finally, the quaternary C4 at 158.3 ppm was confirmed by the cross-peaks that indicated the $^2J_{CH}$ and $^3J_{CH}$ coupling with HN and H5, respectively. It was also possible to observe a slight cross-peak indicating the $^4J_{CH}$ coupling with H8 (Figure S4).

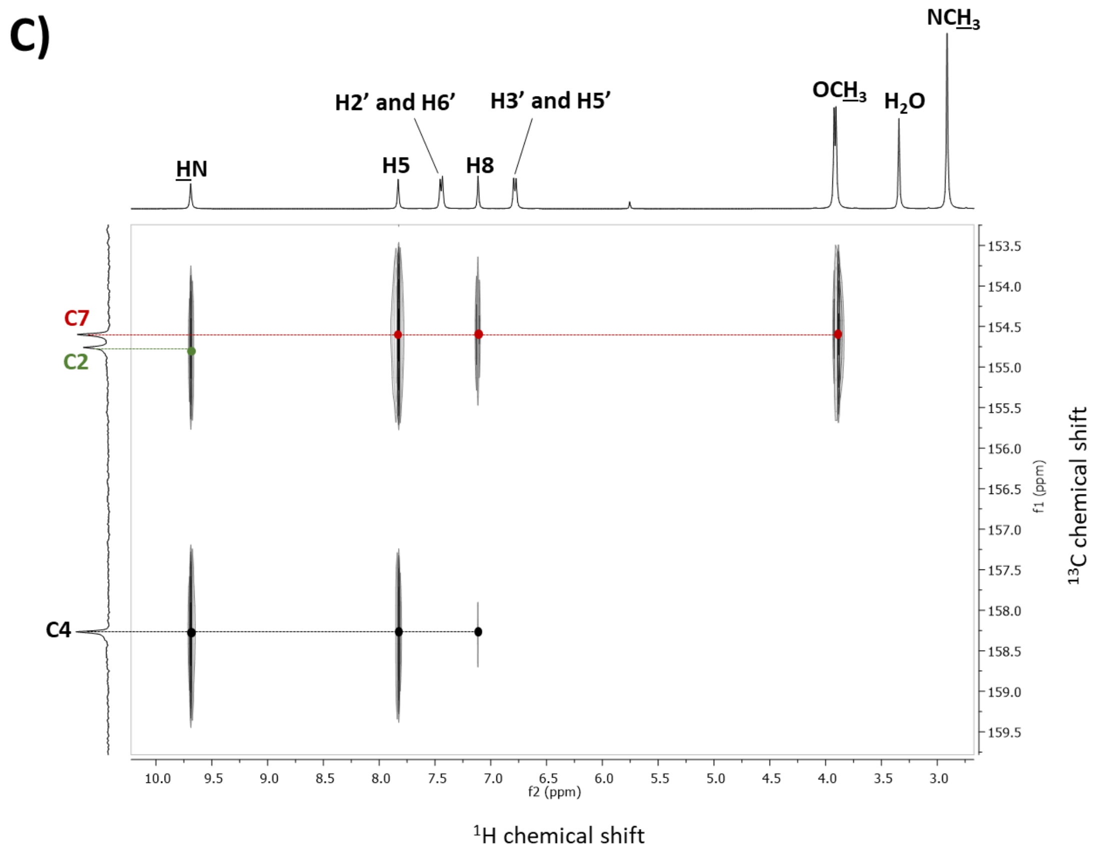

Figure S4. Part C of 2D HMBC NMR of LASSBio-1812 (**21**). C7 (red) showed cross-peaks with H5 and OCH_3 (red line), C2 (green) showed a cross-peak with NH (green line), and C4 (black) showed cross-peaks with NH, H5 and H8 (black line).

References:

1- Barbosa, M.L.d.C.; Lima, L.M.; Tesch, R.; Sant'Anna, C.M.R.; Totzke, F.; Kubbutat, M.H.G.; Schächtele, C.; Laufer, S.A.; Barreiro, E.J. Novel 2-Chloro-4-Anilino-Quinazoline Derivatives as EGFR and VEGFR-2 Dual Inhibitors. *Eur. J. Med. Chem.* 2014, *71*, 1–14. https://doi.org/10.1016/j.ejmech.2013.10.058.
